# Prevention of Cyclophosphamide-Induced Immunosuppression in Mice with the Antimicrobial Peptide Sublancin

**DOI:** 10.1155/2018/4353580

**Published:** 2018-05-07

**Authors:** Shuai Wang, Shuo Huang, Qianhong Ye, Xiangfang Zeng, Haitao Yu, Desheng Qi, Shiyan Qiao

**Affiliations:** ^1^State Key Laboratory of Animal Nutrition, Beijing Key Laboratory of Biofeed Additives, Ministry of Agriculture Feed Industry Centre, China Agricultural University, Beijing 100193, China; ^2^Department of Animal Nutrition and Feed Science, College of Animal Science and Technology, Huazhong Agricultural University, Wuhan, Hubei 430070, China

## Abstract

Sublancin is a glycosylated antimicrobial peptide produced by *Bacillus subtilis* 168 with combined antibacterial and immunomodulatory activities. The purpose of this study was to evaluate the protective effects of sublancin on immunosuppression in cyclophosphamide-treated mice. In normal mice, the phagocytic activity of mouse peritoneal macrophages was significantly enhanced by oral administration of sublancin (1.0 mg/kg body weight) to BALB/c mice for 7 days (*P* < 0.01). In addition, the mRNA expression of IL-1*β*, IL-6, and TNF-*α* in peritoneal macrophages from sublancin- (1.0 mg/kg body weight) administered mice was significantly increased (*P* < 0.05). In cyclophosphamide-treated mice, oral sublancin administration accelerated the recovery of peripheral white blood cells, red blood cells, hemoglobins, and platelets and enhanced the macrophage phagocytic activity. Furthermore, sublancin restored the mRNA levels of IL-2, IL-4, and IL-6 in the spleen. Finally, the intestinal absorption of sublancin was poor as detected in the Caco-2 transwell system. Taken together, these findings suggest that sublancin plays a crucial role in the protection against immunosuppression in cyclophosphamide-treated mice and could be a potential candidate for use in immune therapy regimens.

## 1. Introduction

Cyclophosphamide (Cy) is a major constituent of cancer chemotherapy agent and widely used in the treatment of various types of cancer [1]. Unfortunately, immunosuppression induced by Cy increases incidence of secondary infections and mortality, which is a major limiting factor in clinical chemotherapy [2]. Therefore, many attempts are being investigated to obtain immunomodulatory agents that can reduce the cytotoxic side effects and enhance immunity in chemotherapy-treated patients.

Antimicrobial peptides (AMPs) are a variety of naturally short-amino-acid-chain molecules that provide immediately effective and nonspecific defenses against invading pathogens [3]. Emerging evidence suggests that AMPs are involved in the modulation of the immune response [4–6]. Sublancin is a 37-amino acid AMP produced by *Bacillus subtilis* 168 with high stability [7]. In addition to direct antibacterial activity, sublancin has been reported to possess immunomodulatory activity [8]. Our previous study indicated that sublancin ameliorated *Clostridium perfringens*-induced necrotic enteritis in broilers via alleviating inflammatory reaction [9]. In addition, it was demonstrated that sublancin attenuated the intestinal inflammation induced by methicillin-resistant *Staphylococcus aureus* (MRSA) in mice through inhibition of NF-*κ*B activation [8]. Moreover, we found that macrophages are critical for the protective effect of sublancin in a mouse model of MRSA-induced sublethal infection (unpublished data).

However, the immunomodulatory effect of sublancin on immunosuppressed mice remains poorly understood. Additionally, whether AMPs can be absorbed by the intestine is rarely reported. The objective of this study was to elucidate the protective effects of sublancin on immunosuppression in Cy-treated mice. In this study, we (i) investigated the effects of oral administration of sublancin on peritoneal macrophages in vivo, (ii) evaluated the protective effects of sublancin in a mouse immunosuppression model, and (iii) studied the intestinal absorption of sublancin in the Caco-2 transwell system as well as in the in vivo biodistribution of sublancin.

## 2. Materials and Methods

### 2.1. Animals

Female BALB/c mice of 6–8 weeks old were purchased from HFK Bioscience Co. Ltd. (Beijing, China). The mice were housed in plastic cages under 12 h light/dark cycle and were provided with food and water ad libitum. All experimental procedures were approved by the China Agricultural University Institutional Animal Care and Use Committee (ID: SKLAB-B-2010-003).

### 2.2. Effects of Oral Administration of Sublancin on Peritoneal Macrophages In Vivo

#### 2.2.1. Experimental Design

Sublancin was produced in our laboratory using a highly efficient expression system involving *Bacillus subtilis* 800. The amino acid sequence of sublancin was GLGKAQCAALWLQCASGGTIGCGGGAVACQNYRQFCR, and the peptide purity was >99.6% as determined by high-performance liquid chromatography. Sublancin was produced as lyophilized powder and stored at –20°C until use. Six-week-old female BALB/c mice (*n* = 6) were orally administered with sublancin at 0.5, 1.0, or 2.0 mg/kg body weight (BW)/d for 7 consecutive days. Mice in the control group were administrated with sterile saline daily. Mice in the positive control group were given 2.5 mg/kg BW/d levamisole hydrochloride (LH) in a similar manner as the sublancin treatment. On day 8, peritoneal macrophages (P-Mac) were collected as previously described [10] and maintained in Dulbecco's modified Eagle's medium (DMEM) supplemented with 10% fetal bovine serum (FBS), 100 U/mL penicillin, and 100 *μ*g/mL streptomycin. Peritoneal macrophages were harvested for phagocytosis assay and determining the gene expression levels of cytokines by real-time PCR.

#### 2.2.2. Phagocytosis Assay

Phagocytosis was determined by flow cytometry as described previously [11]. Briefly, peritoneal macrophages from sublancin-administered mice were cultured in 6-well plates (1 × 10^6^ cells/well). After cultivation for 12 h, 100 *μ*L of suspended fluorescent microspheres in PBS was added to the wells (cell-to-bead ratio 1 : 20) and the cells were incubated at 37°C for 1 h. Phagocytosis was terminated by the addition of 2 mL ice-cold PBS, and then the cells were washed three times with cold PBS and harvested. Flow cytometric analysis was carried out using a FACSCalibur flow cytometer with CellQuest software (BD Biosciences, San Jose, CA, USA).

#### 2.2.3. Quantitative Real-Time PCR

Peritoneal macrophages were inoculated in supplemented DMEM (10% FBS, 100 U/mL penicillin, and 100 *μ*g/mL streptomycin) into 6-well plates at 1 × 10^6^ cells/well and cultured for 12 h. Then, total RNA was extracted from the cell pellet using the TRIzol reagent (Invitrogen, Carlsbad, CA). The quality and quantity of total RNA were determined by gel electrophoresis and a NanoDrop 2000 Spectrophotometer (Thermo Fisher Scientific, Wilmington, DE). The first-strand cDNA was synthesized from the extracted RNA (1 *μ*g) using a PrimeScript 1st Strand cDNA Synthesis Kit (Takara, Otsu, Japan) according to the manufacturer's instructions. Real-time PCR was performed on a LightCycler (Roche) with SYBR Green PCR Master Mix (Takara, Otsu, Japan). The relative amounts of mRNAs were normalized against GAPDH and analyzed using the 2^−ΔΔCt^ method [12]. The primer sequences used were as follows: IL-1*β* forward, 5′-GCCTTGGGCCTCAAAGGAAAGAATC-3′; IL-1*β* reverse, 5′-GGAAGACACAGATTCCATGGTGAAG-3′; IL-6 forward, 5′-TGGAGTCACAGAAGGAGTGGCTAAG-3′; IL-6 reverse, 5′-TGGAGTCACAGAAGGAGTGGCTAAG-3′; TNF-*α* forward, 5′-CCTCCCTCTCATCAGTTCTATGG-3′; TNF-*α* reverse, 5′-CGTGGGCTACAGGCTTGTC-3′; GAPDH forward, 5′-ACCCCAGCAAGGACACTGAGCAAG-3′; and GAPDH reverse, 5′-ACCCCAGCAAGGACACTGAGCAAG3′.

### 2.3. Cyclophosphamide-Induced Immunosuppression in Mice

#### 2.3.1. Experimental Model and Treatment Protocols

Eight-week-old female BALB/c mice were randomly assigned to 6 groups consisting of 9 mice each. Mice in the normal control (NC) group were treated once daily with sterile saline for 10 consecutive days. From days 1 to 3, the other five groups of mice were administered with cyclophosphamide at 80 mg/kg BW/d via intraperitoneal injection. From days 4 to 10, the mice were given the following treatments: the model control (MC) group was gavaged with 0.2 mL sterile saline; the three sublancin groups were gavaged with 0.2 mL sublancin at 2.0, 4.0, and 8.0 mg/kg BW/d; and the positive control group was gavaged with 0.2 mL levamisole hydrochloride (LH) at 10 mg/kg BW/d. Levamisole hydrochloride is an agent that has been previously used as an antihelminthic drug in clinical application. In addition, the immunoenhancing effects of levamisole hydrochloride have been demonstrated by many studies [13, 14]. We chose levamisole hydrochloride as a positive control in a cyclophosphamide-induced immunosuppressed mouse model according to several similar previous studies [15, 16]. Body weight of each animal was measured on days 1, 4, and 11. Twenty-four hours after the last drug administration, the animals were killed by cervical dislocation. Three of the animals in each group were used for the carbon clearance test, and the other six were used for other experiments as described below.

#### 2.3.2. Carbon Clearance Test

The macrophage phagocytic function was assessed via the carbon clearance test. On day 11, mice were injected with diluted India ink through the tail vein, at a concentration of 100 *μ*L/10 g body weight. Blood samples were collected from the retinal venous plexuses at 2 min (*t*_1_) and 10 min (*t*_2_) after the injection, and 20 *μ*L blood was then mixed immediately with 2 mL 0.1% Na_2_CO_3_. The absorbance at 600 nm was measured on a spectrophotometer with 0.1% Na_2_CO_3_ as the blank. The liver and the spleen were weighted, and the rate of carbon clearance (*K*) as well as the carbon clearance index (*α*) was calculated by the following formulas: *K* = (lg OD_1_–lg OD_2_)/(*t*_2_–*t*_1_), $\alpha =\sqrt[{3}]{Κ}\times body\;weight/\left(liver\;weight+spleen\;weight\right)$, *t*_2_ = 10 min, and *t*_1_ = 2 min, where OD_1_ and OD_2_ are the absorbance values at 2 min and 10 min, respectively.

#### 2.3.3. Peripheral Hemogram Analysis

Blood was collected into clean tubes with ethylenediaminetetraacetic acid (EDTA) by extracting the eyeballs on the day of sacrifice. Peripheral hemogram analysis was carried out using a Coulter LH755 Hematology Analyzer, which included white blood cells (WBC), red blood cells (RBC), hemoglobins (HGB), and platelets.

#### 2.3.4. Measurement of Cytokine mRNAs in the Spleen

Twenty-four hours after the last administration, six mice from each group were sacrificed via cervical dislocation, and their spleens were aseptically removed. Total RNA was extracted, and the levels of mRNA expression of IL-2, IL-4, and IL-6 were evaluated by real-time PCR as described above. The following primers were used: IL-2, forward 5′-AGGAACCTGAAACTCCCCAG-3′ and reverse 5′-AAATCCAGAACATGCCGCAG-3′, and IL-4, forward 5′-TCTCGAATGTACCAGGAGCC-3′ and reverse 5′-ACCTTGGAAGCCCTACAGAC-3′.

### 2.4. Caco-2 Cell Culture

The human colon carcinoma cell line Caco-2 was purchased from the American Type Culture Collection (Rockville, MD) and cultured in DMEM supplemented with 10% FBS, 1% nonessential amino acids, 100 units/mL penicillin, and 100 *μ*g/mL streptomycin. Cultures were maintained in a humidified incubator at 37°C, with an atmosphere containing 5% CO_2_. For the transport experiment, subconfluent cells were seeded at a density of 4 × 10^5^ cells per well onto microporous membranes inserted into transwells (0.4 *μ*m pores, 1.13 cm^2^, Corning). The inserts were placed in 12-well plates with 0.5 mL of medium in the apical compartment and 1.5 mL medium in the basolateral compartment. The medium was replaced every other day.

### 2.5. Transport Experiments

The transport experiments were performed 21 days after seeding in medium. The integrity of the Caco-2 cell monolayer was confirmed by examining transepithelial electrical resistance (TEER) using an epithelial tissue voltohmmeter (World Precision Instruments Inc., Florida, USA). Only inserts that exceeded a resistance of 400 Ω·cm^−2^ were used, and the transport experiments were performed with a concentration of 200 *μ*M of sublancin in the apical compartment. After 0, 0.5, 1, 2, and 3 h, 100 *μ*L samples was taken from the basolateral side and 50 *μ*L from the apical side. All samples were stored at −20°C prior to analysis by high-performance liquid chromatography [17]. The apparent permeability coefficient (*P*_app_ in cm/s) was calculated by the formula:

(1) $$\begin{array}{c} P_{app}=\frac{\Delta c\cdot V}{\Delta t\cdot c_{0}\cdot A}, \end{array}$$

where *∆c* is the sublancin concentration (*μ*M) in the receiver compartment, *V* is the volume of the receiver compartment, *∆t* is the duration of the transport experiment (s), *c*_0_ is the initial concentration in the donor compartment (*μ*M), and *A* is the surface area of the membrane (cm^2^).

The cytotoxic effect of sublancin on Caco-2 cells was assayed using the cell counting kit-8 (Sigma-Aldrich). Sublancin showed no cytotoxicity up to a concentration of 1600 *μ*M within 24 h [8].

### 2.6. In Vivo Fluorescence Imaging

To observe the biodistribution of sublancin in vivo, sublancin was labeled with the near-infrared dye Cy7 monoreactive NHS ester (Invitrogen, CA, USA). Female BALB/c mice of 6–8 weeks old were given an oral dose of 100 *μ*L Cy7-stained sublancin or free Cy7. At the indicated time points (0 h, 30 min, 2 h, 6 h, 12 h, 24 h, and 48 h), mice were anesthetized with 2.5% isoflurane and then placed in the in vivo Imaging System (FXPro Kodak). The excitation and emission bandpass filter was 730 nm and 790 nm, respectively. Fluorescence signals were collected by Carestream Molecular Imaging Software. At 12 h, mice were euthanized and the ex vivo optical images of the intestine, heart, liver, spleen, lung, and kidney were recorded using the same system.

### 2.7. Statistical Analysis

Data are expressed as means ± SEM and were analyzed by one-way ANOVA using GLM procedures. Statistical differences among treatments were determined using the Student-Newman-Keuls Multiple Range Test (Prism software, version 5). A *P* value < 0.05 was considered significant.

## 3. Results

### 3.1. Effect of Sublancin on the Phagocytic Activity of Peritoneal Macrophages Ex Vivo

**Figure 1:**
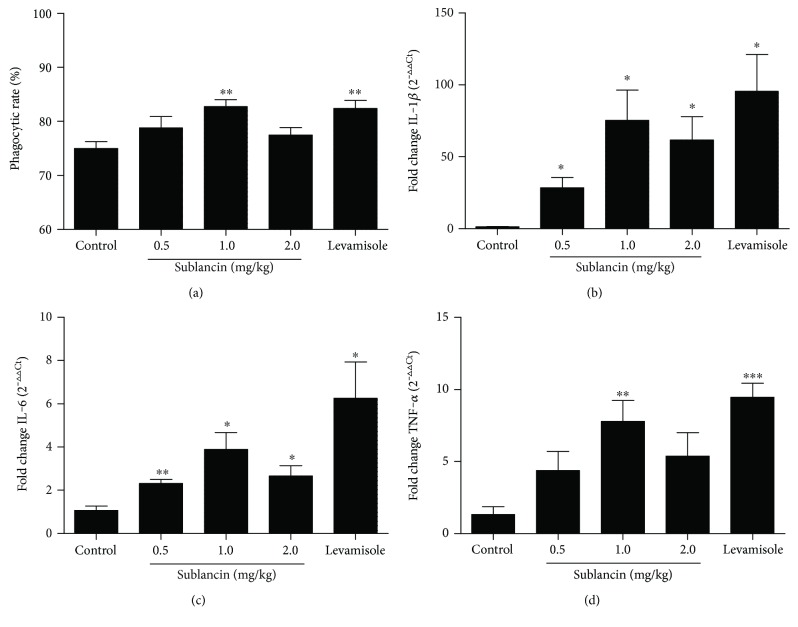
Effect of oral administration of sublancin on the phagocytic activity (a) and gene expression of cytokines (b–d) in P-Mac ex vivo. Six-week-old female BALB/c mice were separated into five groups and orally administered with sublancin (0.5, 1.0, and 2.0 mg/kg body weight/day) or 2.5 mg/kg levamisole hydrochloride (positive control) for 7 days. The vehicle control group was administered with distilled water daily. Mouse P-Mac were isolated and cultured in 10% FBS DMEM. After cultivation, the cells were used to analyze the phagocytic activity and gene expression of cytokines, respectively. Values are expressed as means ± SEM (*n* = 6); ^∗^ *P* < 0.05, ^∗∗^ *P* < 0.01, and ^∗∗∗^ *P* < 0.001 compared with vehicle control group.

In our previous study, we found that sublancin could activate macrophage cell line RAW264.7 cells and peritoneal macrophages in vitro (unpublished data). Therefore, we further investigate the effect of oral administration of sublancin on the phagocytic activity of peritoneal macrophages ex vivo. As shown in Figure 1(a), the sublancin treatment of 1.0 mg/kg significantly augmented the phagocytic activity of peritoneal macrophages ex vivo (*P* < 0.01). These findings are consistent with the results of the phagocytic activity obtained by RAW264.7 cells and peritoneal macrophages in vitro.

### 3.2. Effect of Sublancin on the Gene Expression of Cytokines in Peritoneal Macrophages In Vivo

As indicated in Figures 1(b) and 1(c), oral administration of sublancin for 7 days significantly enhanced the gene expression levels of IL-1*β* and IL-6 in peritoneal macrophages (*P* < 0.05). Additionally, the gene expression level of TNF-*α* was increased significantly in 1.0 mg/kg sublancin treatment compared with control (*P* < 0.05) (Figure 1(d)). For the 2.5 mg/kg levamisole hydrochloride, the gene expression levels of all these three cytokines were also higher than that in the control group (*P* < 0.05).

### 3.3. Effect of Sublancin on the Body Weight in Cyclophosphamide-Treated Mice

**Figure 2:**
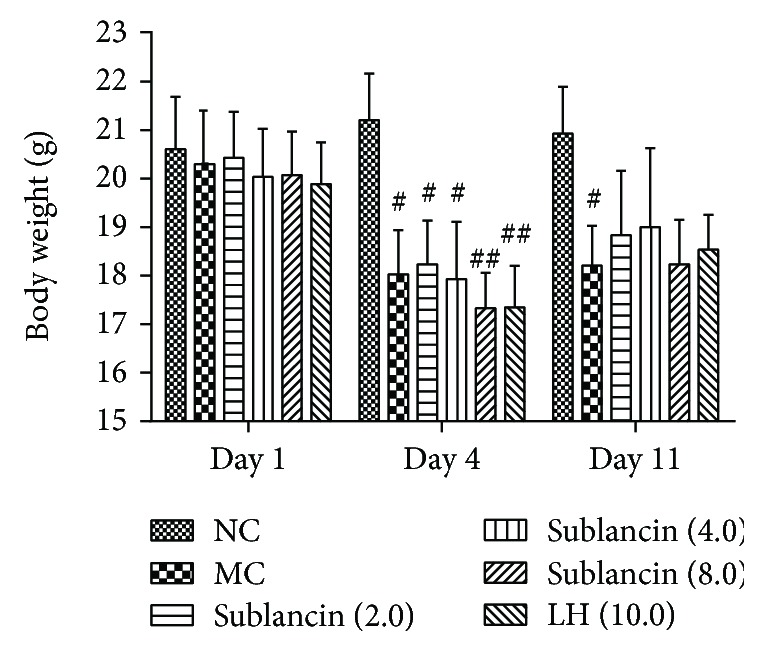
Effects of sublancin on the body weight in cyclophosphamide-treated mice. NC (normal control) = mice were treated with saline; MC (model control) = mice were administered with cyclophosphamide 80 mg/kg/d via i.p. injection for 3 days and gavaged with saline for 7 days; sublancin (2.0, 4.0, and 8.0) = mice were administered with cyclophosphamide 80 mg/kg/d via i.p. injection for 3 days and gavaged with sublancin at 2.0, 4.0, or 8.0 mg/kg/d for 7 days, respectively; LH (10.0) = mice were administered with cyclophosphamide 80 mg/kg/d via i.p. injection for 3 days and gavaged with levamisole hydrochloride for 7 days. Values are expressed as means ± SEM (*n* = 9); ^#^ *P* < 0.05 and ^##^ *P* < 0.01 compared with NC group.

In order to evaluate the immunomodulatory effects of sublancin, a cyclophosphamide-induced immunosuppressed mouse model was established in this study. The body weight of mice in each treatment is summarized in Figure 2. Prior to the cyclophosphamide injection (day 1), the body weight did not differ (*P* > 0.05) among the treatment groups. All mice that were injected with cyclophosphamide exhibited a significant decrease in body weight compared with the normal control mice (*P* < 0.05). Twenty-four hours after the last drug administration (day 11), body weights were still poorer in the model control than in the normal control. Treatment with all 3 sublancin levels resulted in similar body weight to levamisole hydrochloride.

### 3.4. Sublancin Activates the Phagocytic Activity of the Macrophage System

**Figure 3:**
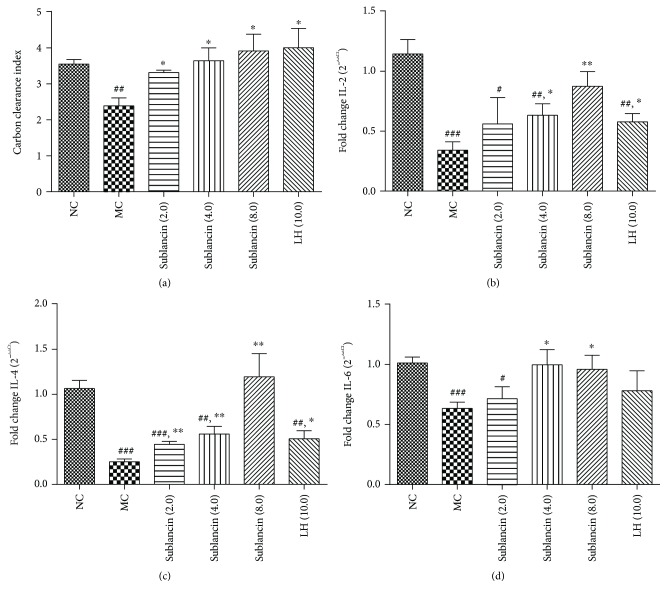
Effect of sublancin on the macrophage phagocytic index employing the carbon clearance test (a) and cytokine mRNA expression in the spleen of immunosuppressed mice (b–d). NC (normal control) = mice were treated with saline; MC (model control) = mice were administered with cyclophosphamide 80 mg/kg/d via i.p. injection for 3 days and gavaged with saline for 7 days; sublancin (2.0, 4.0, and 8.0) = mice were administered with cyclophosphamide 80 mg/kg/d via i.p. injection for 3 days and gavaged with sublancin at 2.0, 4.0, or 8.0 mg/kg/d for 7 days, respectively; LH (10.0) = mice were administered with cyclophosphamide 80 mg/kg/d via i.p. injection for 3 days and gavaged with levamisole hydrochloride for 7 days. Values are expressed as means ± SEM of three (a) or six (b–d) animals. ^#^ *P* < 0.05, ^##^ *P* < 0.01, and ^###^ *P* < 0.001 compared with NC group. ^∗^ *P* < 0.05 and ^∗∗^ *P* < 0.01 compared with MC group.

The carbon clearance test was performed to investigate the effect of sublancin on macrophage phagocytic activity. As shown in Figure 3(a), the carbon clearance index *α* in cyclophosphamide-treated mice was significantly lower than that in the normal control (*P* < 0.01). However, the inhibitory effect of cyclophosphamide was significantly alleviated by sublancin. Mice in the three sublancin level treatments (2.0, 4.0, and 8.0 mg/kg) had higher carbon clearance index *α* values than those in the model control, indicating that sublancin can enhance the phagocytic activity of the reticuloendothelial system.

### 3.5. Effect of Sublancin on the Cytokine mRNA Expression in Cyclophosphamide-Treated Mice

It was found that cyclophosphamide treatment reduced the IL-2, IL-4, and IL-6 mRNA expression levels significantly in the MC group, compared with the NC mice as shown in Figure 3 (*P* < 0.001). As compared to the MC group, mice treated with sublancin at doses of 4.0 mg/kg and 8.0 mg/kg showed a significant increase in the mRNA levels of IL-2, IL-4, and IL-6 (*P* < 0.05).

### 3.6. Effect of Sublancin on Hemopoietic Function in Cyclophosphamide-Treated Mice

**Figure 4:**
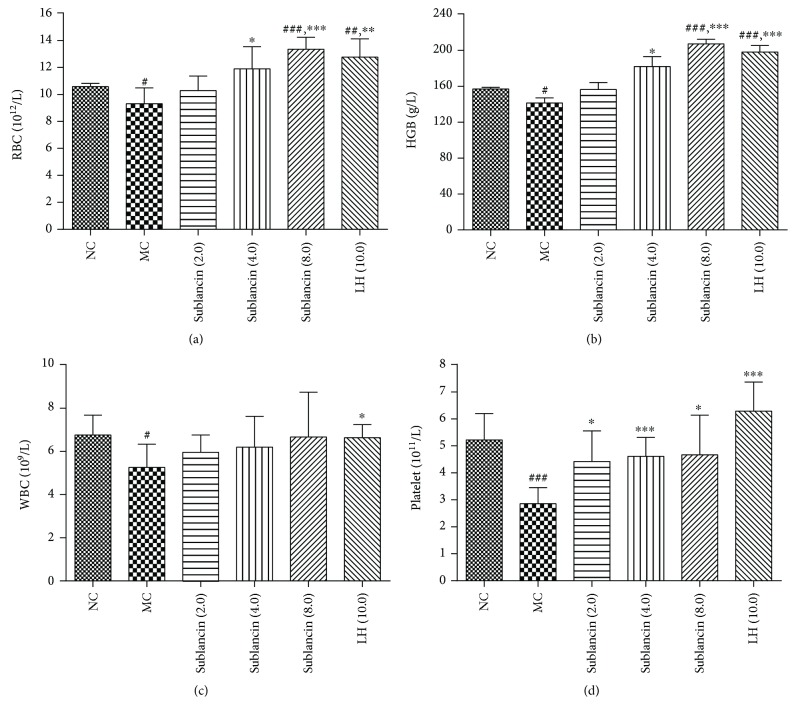
Effects of sublancin on numbers of red blood cells (RBC, 10^12^/L) (a), hemoglobin (HGB, g/L) (b), white blood cells (WBC, 10^9^/L) (c), and platelet (10^11^/L) (d) in cyclophosphamide-treated mice. NC (normal control) = mice were treated with saline; MC (model control) = mice were administered with cyclophosphamide 80 mg/kg/d via i.p. injection for 3 days and gavaged with saline for 7 days; sublancin (2.0, 4.0, and 8.0) = mice were administered with cyclophosphamide 80 mg/kg/d via i.p. injection for 3 days and gavaged with sublancin at 2.0, 4.0, or 8.0 mg/kg/d for 7 days, respectively; LH (10.0) = mice were administered with cyclophosphamide 80 mg/kg/d via i.p. injection for 3 days and gavaged with levamisole hydrochloride for 7 days. Values are expressed as means ± SEM (*n* = 6); ^#^ *P* < 0.05, ^##^ *P* < 0.01, and ^###^ *P* < 0.001 compared with NC group. ^∗^ *P* < 0.05, ^∗∗^ *P* < 0.01, and ^∗∗∗^ *P* < 0.001 compared with MC group.

In order to evaluate the protective effect of sublancin on the myelosuppression induced by sublancin, the RBC, WBC, HGB, and platelet numbers from peripheral blood were analyzed. As shown in Figure 4, peripheral RBC, WBC, HGB, and platelet counts were significantly decreased (*P* < 0.05) in the MC mice compared with the NC mice. Mice treated with 4.0 mg/kg and 8.0 mg/kg sublancin had higher (*P* < 0.05) RBC, HGB, and platelets than mice in the MC group. A numerical increase in the number of WBC was observed in the three sublancin treatments compared with the MC group. In addition, treatment with 10.0 mg/kg levamisole hydrochloride resulted in higher numbers of RBC, WBC, HGB, and platelets compared with the NC treatment. These findings indicated that sublancin can ameliorate myelosuppression induced by cyclophosphamide.

### 3.7. Intestinal Absorption of Sublancin

The intestinal absorption of sublancin was evaluated using the Caco-2 transwell system.  Table 1 summarizes the apical and basolateral concentrations of sublancin and the calculated *P*_app_ at different time points of the transport experiment. After 3 h of incubation, 23.59 ± 1.96 *μ*M of sublancin was detected on the basolateral side, corresponding with a *P*_app_ of 9.58 ± 0.58 × 10^−6^ cm·s^−1^. It has been reported that compounds with poor absorption have *P*_app_ values less than 10 × 10^−6^ cm·s^−1^ [18]. The results from the current study indicated that sublancin was poorly absorbed by the Caco-2 monolayer.

### 3.8. In Vivo Biodistribution of Sublancin

**Figure 5:**
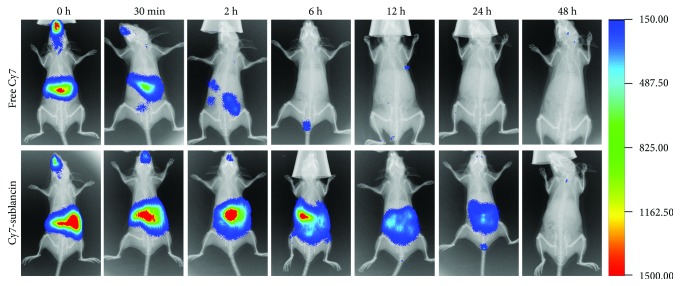
In vivo biodistribution of Cy7-labeled fluorescent sublancin in 6–8-week-old BALB/c mice evaluated by near-infrared fluorescent scans.

**Figure 6:**
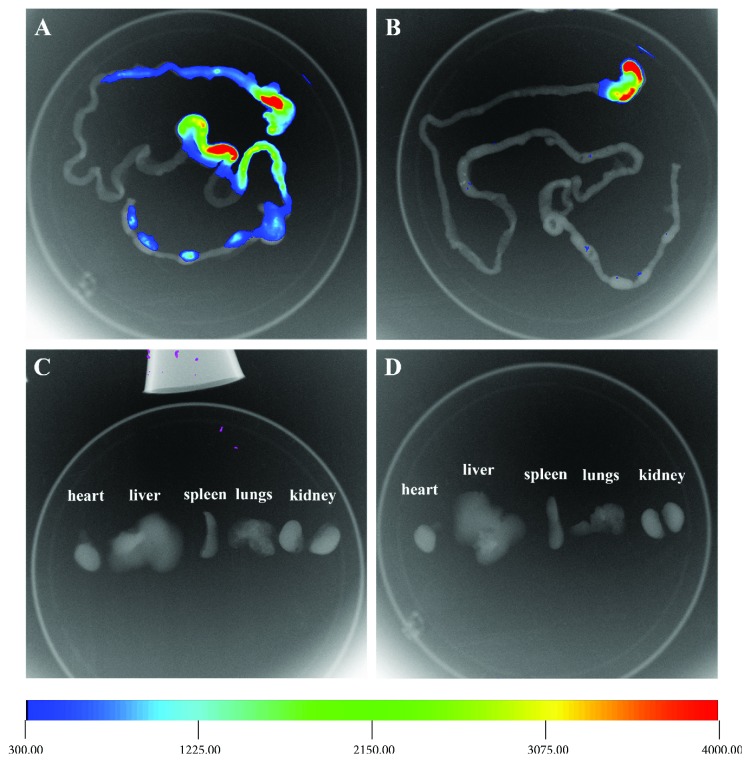
The ex vivo optical images of the intestines and organs of 6–8-week-old BALB/c mice with oral administration of Cy7-labeled fluorescent sublancin at 12 h. Monitor of the Cy7-labeled sublancin in the excised intestines (a) and organs (c); monitor of the free Cy7 in the excised intestines (b) and organs (d).

To investigate the in vivo biodistribution of sublancin, the sublancin labeled with Cy7 was given orally to 6–8-week-old female BALB/c mice. As shown in Figure 5, Cy7-stained sublancin was continuously retained in the mouse intestine 24 h after the oral administration. In contrast, mice gavaged with free Cy7 showed weak fluorescent signals within 6 h. Twelve hours after administration, most of the free Cy7 had disappeared from the intestine. In addition, 12 h after gavage, the mice were euthanized and the intestine, heart, liver, spleen, lung, and kidney were excised to obtain ex vivo fluorescent images. As shown in Figure 6(a), a large amount of fluorescent Cy7-stained sublancin was continuously retained in the intestine. However, only very little of fluorescent free Cy7 was still located in the intestine. Almost no fluorescent signal was detected in the heart, liver, spleen, lung, and kidney of mice gavaged with Cy7-stained sublancin or free Cy7.

## 4. Discussion

The immune system plays a crucial role in the treatment of many diseases during chemotherapeutic intervention [19]. Emerging evidence indicates that modulation of immune response provides distinct advantages over conventional therapies [20, 21]. Macrophages are professional phagocytes for host defense against infections. Activated macrophages exhibit the ability to enhance phagocytosis and cytokine production [22]. It has been reported that orally administered AMPs could confer protection by immune modulation. Oral Pep19-2.5 (a synthetic AMP) administration was demonstrated to improve the clinical symptoms in *Salmonella typhimurium*-infected mice due to its ability to modulate immunity [23]. The AMP cecropin AD protected weaned piglets against *Escherichia coli* infection via increasing immune status [24]. Here, we show that oral administration of sublancin to BALB/c mice for 7 days enhanced phagocytic activity of peritoneal macrophages and promoted the mRNA expression of IL-1*β*, IL-6, and TNF-*α* by peritoneal macrophages. Additionally, a lack of change in the total number of peritoneal cells or other immune cell subset was observed after exposure of mice to various doses of sublancin (Figure S1). These observations suggest that sublancin could effectively activate macrophages and consequently improve the innate immunity.

It is well accepted that cyclophosphamide is an important chemotherapeutic drug in tumor treatment. However, cyclophosphamide also has adverse effects on healthy cells and results in immunosuppression [25]. In the present study, mice treated with cyclophosphamide were used as an immunosuppression model. Cyclophosphamide treatment resulted in a significant decrease in body weight. However, no difference was observed in the body weight among the sublancin-treated groups and the MC group on day 11, a result similar to the effects of levamisole hydrochloride.

Phagocytes (neutrophils, monocytes, and macrophages) are important actors in the innate immune response and are also the earliest cell types in response to pathogen invasions [26]. Macrophages are the most important phagocytes, and they play a pivotal role in host defense against diverse types of invasive cells, such as tumor cells [27]. Using immunomodulators for improving antitumor functions of macrophages is an area with good prospect for cancer chemotherapy [28]. The function of macrophages was evaluated with the carbon clearance test. Our results indicate that cyclophosphamide treatment impaired phagocytic activity of the macrophage system, which is consistent with previous reports [29, 30]. However, oral sublancin administration substantially enhanced the phagocytic activity of the reticuloendothelial system in immunosuppressed mice, indicating that sublancin improved innate immune function of immunosuppressed mice.

Myelosuppression induced by cyclophosphamide administration is a major problem for cancer patients in clinical chemotherapy. In agreement with the results of previous studies [31, 32], cyclophosphamide treatment markedly decreased the count of peripheral RBC, WBC, HGB, and platelets. We observed that sublancin restored peripheral RBC, WBC, HGB, and platelet counts, suggesting that sublancin could protect against myelosuppression induced by cyclophosphamide.

T lymphocytes play an important role in recognizing and presenting antigens. IL-2 is a Th1-derived cytokine that is crucial for cell-mediated immune responses. IL-4 and IL-6 are produced by Th2 cells and can promote humoral immunity [33]. It was found that sublancin treatment had no significant effects on the total number of splenic cells and immune cell subset in the spleen (Figure S1). In the current study, it showed that the mRNA expression levels of IL-2, IL-4, and IL-6 in the spleen were significantly higher in the sublancin-treated groups (4.0 and 8.0 mg/kg) than in the cyclophosphamide-treated group, suggesting that sublancin could facilitate the recovery of the mRNA expression levels of IL-2, IL-4, and IL-6 in the spleen and alleviate the severity of immunosuppression.

To date, the mechanism of action of AMPs has been well studied and established, but most naturally occurring AMPs are abandoned during the development phase due to their potential lability to proteases or limited bioavailability [34]. The gastrointestinal absorption characteristic is a key issue in early drug development. At present, the half-life of AMPs in the gut after oral administration or whether they can be absorbed by the intestine is poorly reported [35]. Confluent Caco-2 monolayers, cultured on permeable supports, are commonly used to predict the intestinal absorption of drugs [36]. Compounds which are poorly absorbed in the human intestine had *P*_app_ < 10 × 10^−6^ cm·s^−1^ in the Caco-2 transwell system, and compounds with complete absorption had *P*_app_ > 70 × 10^−6^ cm·s^−1^ [18]. According to the abovementioned correlations, sublancin showed poor absorption with a *P*_app_ of 9.58 ± 0.58 × 10^−6^ cm·s^−1^ after 3 hours of incubation. In a previous study, the antimicrobial peptide sublancin did not show any signs of cytotoxicity in the Caco-2 cells as determined by the CCK-8 assay [8], indicating that the Caco-2 cell monolayer may not compromised by sublancin. In addition, we also determined the sublancin concentration in the cell lysate. No sublancin was detected in the cells (data not shown). Hence, we speculate that the observed poor flux might be caused by the paracellular transport. Next, we observed the in vivo biodistribution of Cy7-stained sublancin using the in vivo Imaging System. We found that Cy7-stained sublancin continuously retained in the intestine 24 h after oral administration, whereas mice orally administered with free Cy7 showed weak fluorescent signals within 6 h. This result indicated that Cy7-stained sublancin stayed longer in the intestine than free Cy7. Moreover, almost no fluorescent signal was observed in the heart, liver, spleen, lung, and kidney of mice gavaged with Cy7-stained sublancin 12 h after administration, suggesting that sublancin may not be absorbed into the circulation system by gavage within 12 h. It has been reported that sublancin has an extraordinary high chemical stability, tolerating both low and high pH, as well as gastrointestinal digestive enzymes [7, 37]. We speculate that sublancin and the fluorophore were excreted when the fluorescent signal was no longer detected in the intestine.

## 5. Conclusion

In conclusion, our study demonstrates the immunostimulatory properties of sublancin, which effectively activated macrophages and consequently improved the innate immunity of mice in vivo. Further, oral sublancin administration accelerated the recovery of immunosuppression in cyclophosphamide-treated mice. All these results indicate that sublancin is a potential candidate for use in immune therapy regimens.

## Figures and Tables

**Table 1:** Summary of the analysis of sublancin through a Caco-2 monolayer.

Incubation time (h)	*C*_apical_ (*μ*M)	*C*_basolateral_ (*μ*M)	*P*_app_ (10^−6^ cm·s^−1^)
0.5	147.4 ± 1.48	<LOD	—
1	135.7 ± 3.37	3.78 ± 1.02	7.00 ± 0.25
2	113.5 ± 4.20	9.56 ± 2.21	7.65 ± 0.69
3	112.0 ± 2.27	23.59 ± 1.96	9.58 ± 0.58

The concentrations determined by high-performance liquid chromatography are shown as mean ± SEM (*n* = 6). Based on these data, the transport rate *P*_app_ was calculated.

## Data Availability

The data used to support the findings of this study are available from the corresponding author upon request.

## Supplementary Materials

Supplementary Materials Figure S1: effects of sublancin on the immune cell subset in the peritoneal cavity (PerC) and spleen in vivo. BALB/c mice were orally administered with sublancin for 28 days (0.3 mg/kg and 1.0 mg/kg) or 14 days (0.6 mg/kg and 1.2 mg/kg), and the peritoneal cells and spleen were collected 24 hours after the last dose for flow cytometry. (A and B) A lack of change in the total number of peritoneal cells and splenic cells was observed after exposure of mice to various doses of sublancin. (C) Comparison of the mean percentage and cell numbers of peritoneal myeloid cells (CD 11b+), B cells (B220+), macrophages (F4/80+), and neutrophils (Ly6G+) in the peritoneal cavity between sublancin and control mice. (D) The average percentage and cell numbers of CD4+ T cells (CD4+), CD8+ T cells (CD8+), B cells (B220+), and NK cells (NK1.1+) in the spleen of sublancin and control mice are compared. Values are expressed as means ± SEM.
