# Supplementary figures and images for: Prevention of Cyclophosphamide-Induced Immunosuppression in Mice with the Antimicrobial Peptide Sublancin
Source: J Immunol Res. 2018 May 7;2018:4353580. doi: 10.1155/2018/4353580 (PMC5964538; doi:10.1155/2018/4353580)

Figure S1


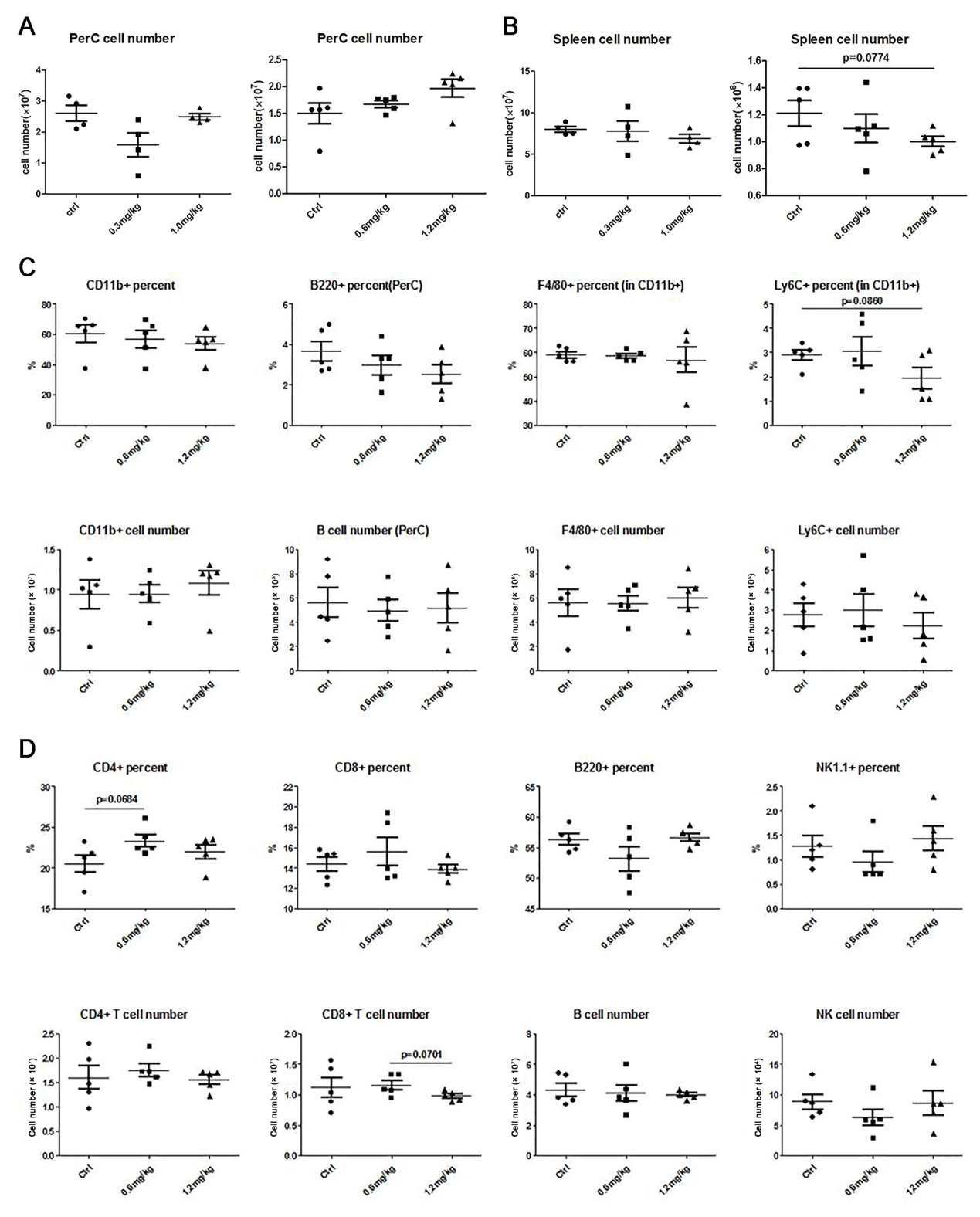

Supplement: Supplementary Materials — Figure S1: effects of sublancin on the immune cell subset in the peritoneal cavity (PerC) and spleen in vivo. BALB/c mice were orally administered with sublancin for 28 days (0.3 mg/kg and 1.0 mg/kg) or 14 days (0.6 mg/kg and 1.2 mg/kg), and the peritoneal cells and spleen were collected 24 hours after the last dose for flow cytometry. (A and B) A lack of change in the total number of peritoneal cells and splenic cells was observed after exposure of mice to various doses of sublancin. (C) Comparison of the mean percentage and cell numbers of peritoneal myeloid cells (CD 11b+), B cells (B220+), macrophages (F4/80+), and neutrophils (Ly6G+) in the peritoneal cavity between sublancin and control mice. (D) The average percentage and cell numbers of CD4+ T cells (CD4+), CD8+ T cells (CD8+), B cells (B220+), and NK cells (NK1.1+) in the spleen of sublancin and control mice are compared. Values are expressed as means ± SEM. [file 4353580.f1.docx]
